# Help seeking behavior and onset-to-alarm time in patients with acute stroke: sub-study of the preventive antibiotics in stroke study

**DOI:** 10.1186/s12883-016-0749-2

**Published:** 2016-11-25

**Authors:** E. Zock, H. Kerkhoff, R. P. Kleyweg, T. B. V. van Bavel-Ta, S. Scott, N. D. Kruyt, P. J. Nederkoorn, D. van de Beek

**Affiliations:** 1 Department of Neurology, Albert Schweitzer Hospital Dordrecht, Albert Schweitzerplaats 25, 3318 AT Dordrecht, The Netherlands; 2 Department of Neurology, St. Elisabeth hospital Tilburg, Tilburg, The Netherlands; 3 Department of Neurology, Slotervaart Hospital Amsterdam, Amsterdam, The Netherlands; 4 Department of Neurology, Leiden University Medical Center, Leiden, The Netherlands; 5 Department of Neurology, Academic Medical Center Amsterdam, Amsterdam, The Netherlands

**Keywords:** Stroke, Illness Behavior, Health Knowledge, Attitudes, Practice, Time-to Treatment, Thrombolytic Therapy

## Abstract

**Background:**

Patients with acute stroke often do not seek immediate medical help, which is assumed to be driven by lack of knowledge of stroke symptoms. We explored the process of help seeking behavior in patients with acute stroke, evaluating knowledge about stroke symptoms, socio-demographic and clinical characteristics, and onset-to-alarm time (OAT).

**Methods:**

In a sub-study of the Preventive Antibiotics in Stroke Study (PASS), 161 acute stroke patients were prospectively included in 3 Dutch hospitals. A semi-structured questionnaire was used to assess knowledge, recognition and interpretation of stroke symptoms. With in-depth interviews, response actions and reasons were explored. OAT was recorded and associations with socio-demographic, clinical parameters were assessed.

**Results:**

Knowledge about stroke symptoms does not always result in correct recognition of own stroke symptoms, neither into correct interpretation of the situation and subsequent action. In our study population of 161 patients with acute stroke, median OAT was 30 min (interquartile range [IQR] 10–150 min). Recognition of one-sided weakness and/or sensory loss (*p* = 0.046) and adequate interpretation of the stroke situation (*p* = 0.003), stroke at daytime (*p* = 0.002), severe stroke (*p* = 0.003), calling the emergency telephone number (*p* = 0.004), and transport by ambulance (*p* = 0.040) were associated with shorter OAT.

**Conclusion:**

Help seeking behavior after acute stroke is a complex process. A shorter OAT after stroke is associated with correct recognition of one-sided weakness and/or sensory loss, adequate interpretation of the stroke situation by the patient and stroke characteristics and logistics of stroke care, but not by knowledge of stroke symptoms.

**Electronic supplementary material:**

The online version of this article (doi:10.1186/s12883-016-0749-2) contains supplementary material, which is available to authorized users.

## Background

In patients with acute stroke, intravenous thrombolysis with recombinant tissue plasminogen activator and endovascular treatment are effective, but only within 6 h after stroke onset [1, 2]. Unfortunately, many patients with acute stroke do not seek immediate medical help [3, 4]. This knowledge has prompted several public campaigns to increase public awareness of stroke symptoms. However, these campaigns have limited or no sustained effect on the proportion of patients eligible for acute stroke treatments [5–8].

It has been presumed that in case of a stroke situation the patient or bystander has to recognize stroke symptoms, attribute these symptoms to acute stroke, but also has to recognize the importance to seek immediate help [9]. Other factors of importance in the process of help seeking are age, stroke severity and ethnicity [10–12]. The interactions between these factors are unknown. We explored the process of help seeking behavior in patients with acute stroke, evaluating knowledge about stroke symptoms, socio-demographic and clinical characteristics, and onset-to-alarm time (OAT).

## Methods

### Patients

The current prospective observational study was performed as a sub-study of the Preventive Antibiotics in Stroke Study (PASS) [13]. In this multicenter, prospective, randomized, open-label, masked endpoint trial including 2550 patients the clinical benefit of ceftriaxone versus standard stroke care was investigated. Details of the protocol were described previously [14]. In short, eligible patients were aged 18 years or older, had a stroke since less than 24 h and a score of 1 or more on the National Institutes of Health Stroke Scale (NIHSS). For the current sub-study we recruited consecutive patients from 3 participating centers from Nov-1–2011 to May-1–2014. Patients unable to comprehend interviews due to aphasia or language barrier were excluded.

### Methods

Informed consent for this sub-study was obtained and patients were interviewed within 72 h after admission using a questionnaire with 23 closed and open-ended questions based on a pilot study (Additional file 1) [4]. Hospitals were selected on their geographical representation and ability to invest extra time for our questionnaire. Patients were selected on their physical state and the ability to understand and answer the questions of the questionnaire. Knowledge of stroke symptoms was evaluated twofold. Firstly, in open-ended questions patients had to name any stroke signs. Secondly, five symptoms were verbally presented and patients were asked to recall which of these could be signs of stroke. These five symptoms were one-sided weakness and/or sensory loss, any speech disturbance, vision loss with one or both eyes, dizziness and a severe, unusual headache. Knowledge of the three major stroke symptoms together, usually used in stroke campaigns, was also investigated: knowledge of one-sided weakness and/or sensory loss, face asymmetry and speech disturbance. In an open-ended question we explored knowledge about stroke treatment options. Patients were asked whether a therapy for stroke exists, and if yes, what kind of therapy. Exploration of help seeking behavior was subcategorized by recognition, interpretation and action. Recognition was defined as being aware of body cues and realizing that something was wrong and was determined by asking patients which symptoms had been experienced [9]. Interpretation was defined as interpreting symptoms in terms of a disease [9]. Action was defined as the first action taken within one hour after symptom onset (i.e., contacting family, the general practitioner, the emergency medical number or otherwise); if no action was undertaken, reasons were explored. Help seeking behavior was defined as the interaction between knowledge, recognition and interpretation leading to the presence or absence of any action.

Factors previously associated with patient delay (socio-demographic and clinical stroke characteristics, mode of transport) were obtained prospectively. OAT was defined as the time interval between the first moment patients or bystander(s) witnessed symptoms to first action of help seeking. Stroke severity was defined by the National Institute of Health Stroke Scale (NIHSS)-score, categorized in 4 severities, because of low frequencies of the higher scores (defined as minor with a score 1 to 4, moderate 5 to 15, moderate to severe 16 to 20 and severe >20) [15]. Daytime was defined as hours between 6:01 am and 22:59 pm.

### Statistical analysis

Descriptive statistics were used to describe the results of semi-structured and in-depth interviews. Non-parametric tests for dichotomous (Mann Whitney U) and categorical (Kruskal Wallis) variables were used to explore the associations between OAT and socio demographic, clinical characteristics, knowledge and the abovementioned elements of help seeking behavior. A *p* value < 0.05 was considered statistically significant.

## Results

### Baseline characteristics

Between November 1^st^, 2011, and May 1^st^, 2014, 161 patients with acute stroke were included. Not all patients were able to answer all questions. Socio-demographic and clinical characteristics are presented in Table 1. Mean age was 72 years and 53% were men. The majority of patients had a cerebral infarction (90%) and stroke severity was relatively mild with a median NIHSS score of 4 (IQR 3–6). There were no differences in clinical characteristics between patients included in the 3 hospitals (data not shown).

**Table 1:** Socio-demographic and clinical characteristics

Characteristic	No./No. evaluated (%)	Characteristic	No./No. evaluated (%)
Male	85/161 (53)	Time of stroke onset known	
Mean age (yrs)	72	Yes	119/160 (74)
Median pre-stroke mRS (IQR)	0 (0–1)	No	16/160 (10)
Median NIHSS (IQR)	4 (3–6)	Wake up	25/160 (16)
1–4	102/161 (63)	Symptom onset at	
5–15	49/161 (30)	Work	7/161 (4)
16–20	9/161 (6)	Home	126/161 (78)
>20	1/161 (1)	Other	28/161 (17)
History		Diagnosis at admission	
Stroke	53/161 (33)	Ischemic stroke	145/161 (90)
Smoking	93/161 (58)	Haemorrhage	12/161 (8)
Hypertension	81/161 (50)	TIA	4/161 (3)
Diabetes	33/161 (21)	Stroke localization	
Alcoholism	1/161 (1)	Left hemisphere	69/161 (43)
Living together	102/157 (65)	Right hemisphere	73/161 (45)
Bystander present	101/160 (63)	Posterior circulation	17/161 (11)

*mRS* modified Rankin Scale, *IQR* Inter Quartile Range, *NIHHS* National Institute of Health Stroke Scale, *TIA* Transient Ischemic Attack

### Knowledge, recognition and interpretation

Most common knowledge about stroke symptoms was one-sided weakness and/or sensory loss (82 of 158 [52%]), speech disturbance (60 of 158 [38%]) and facial asymmetry (55 of 158 [35%]) (Table 2). Three or more symptoms were simultaneously mentioned by 53 of 159 patients (33%) and 36 of 159 (22%) did not know any stroke symptom. One hundred of 160 patients (62%) could recall three or more symptoms; 8 of 160 patients (5%) could not recall any symptom. Only 16 of 159 patients (10%) knew the three major stroke criteria, one-sided weakness and/or sensory loss, face asymmetry and speech disturbance. Knowledge about treatment options for was present in 77 of 161 patients (48%); suggested treatment options ranged from medication for influencing blood clotting in general sense (18 of 77 [23%]), rehabilitation (16 of 77 [21%]), acute intravenous medication (8 of 77 [10%]) to for example resting and drinking water (3 of 77 [4%]).

**Table 2:** Knowledge

Knowledge of stroke symptoms (open-ended)	No./no. evaluated (%)	Knowledge of stroke symptoms (recall)	No./no. evaluated (%)
One-sided weakness and/or sensory loss	82/158 (52%)	One-sided weakness and/or sensory loss in arm, leg, or face	127/160 (79%)
Face asymmetry	55/158 (35%)	–	
Any speech disturbance	60/158 (38%)	Any speech disturbance	59/158 (37%)
Decreased consciousness	20/158 (13%)	–	
Vision loss	19/158 (12%)	Vision loss one or both eyes	63/160 (39%)
Headache	17/158 (11%)	Severe, unusual headache	71/160 (44%)
Confusion	14/158 (9%)	–	
Dizziness	10/158 (6%)	Dizziness	77/160 (48%)
Nausea/vomiting	7/158 (4%)	–	

Recognition of one’s own one-sided weakness and/or sensory loss and speech disturbance was present in 106 of 158 patients (66%) and 59 of 158 patients (37%) patients respectively. Thirty-three of 157 patients (21%) could not specify the stroke symptom(s) they had experienced (Additional file 2: Table S1) but managed to describe the impact on performance, such as ‘unable to drive a car’ or ‘loss of control’.

Correct interpretation of the stroke situation at symptom onset was made by 46 of 161 patients (29%). However, 66 of 161 patients (41%) had no clue which medical condition caused their symptoms. Another explanation than stroke was given in 49 of 161 patients (30%), such as stress, a heart problem, hypoglycemia, or eye infection.

### Help seeking behavior

**Fig. 1:**
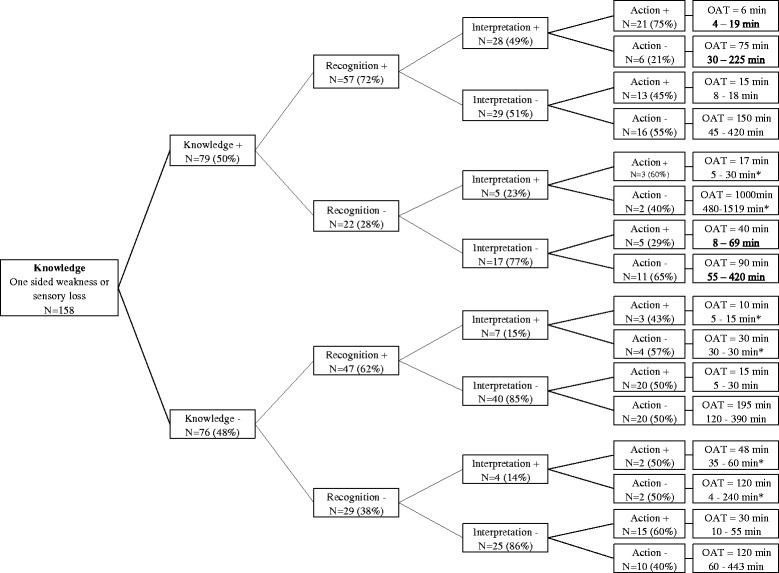
Process of help seeking behavior

The elements of help seeking behavior are visualized in Fig. 1 in patients with and without knowledge about one-sided weakness or sensory loss as stroke warning sign. Fifty-seven of 79 patients (73%) with knowledge about this symptom recognized that something was wrong when they experienced these symptoms themselves. Twenty-eight of these patients (49%) interpreted this as a stroke. When interpreted as a stroke, 21 patients (75%) undertook action within the first hour after symptom onset. In the group of patients without knowledge about stroke symptoms (48%), who did not recognize this symptom (38%) and without a correct interpretation (86%), more than half (60%) undertook action within the first hour after symptom onset. The process for other symptoms as speech disturbance and facial asymmetry showed similar results (Additional file 3: Figure S1).

### Action and onset-to-alarm time

**Fig. 2:**
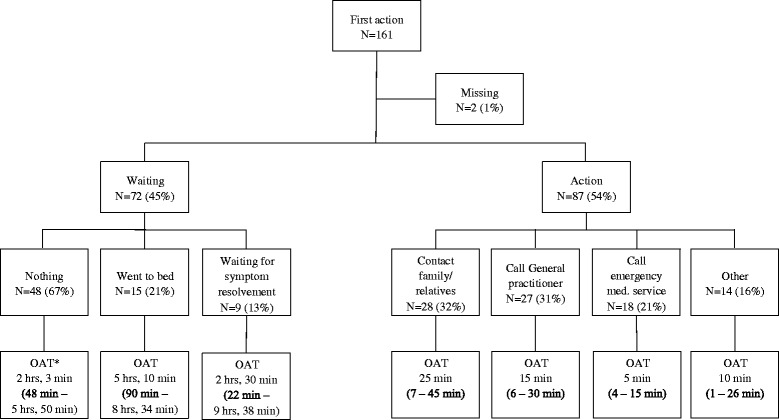
First action and OAT

Action was undertaken in 87 of 161 patients (54%) within the first hour after symptom onset (Fig. 2). The median OAT was 30 min (interquartile range [IQR] 10–150 min). Many of them contacted a relative or friend (28 of 87 [32%]; median OAT 25 min.) or the general practitioner (27 of 87 [31%]; median OAT 15 min.). The emergency medical service was called in 18 of 87 (21%; median OAT 5 min.) of these patients. Some patients (14 of 87 [16%]; median OAT 10 min.) described a combination of several actions, for example, finishing work, going home and consulting family. In some cases family undertook action after for example accidentally calling the patient and noticing that something was wrong. No direct action was undertaken by 72 of 161 patients (45%): many did nothing at all (48 of 72 [67%]; median OAT 2 h, 30 min.), others went to bed (15 of 72 [21%]; median OAT 5 h, 10 min.) or waited for evolvement of symptoms (9 of 72 [13%]; median OAT 2 h, 30 min.). Reasons for not seeking help were: no health problem present (30 of 72 [42%]) or pre-assumption that symptoms would disappear spontaneously (18 of 72 [25%]; data not shown).

Univariate analysis showed that more severe stroke (*p* = 0.003), daytime occurrence of symptoms (median time 30 vs. 270 min; *p* = 0.002), calling the emergency telephone number (*p* = 0.004), and transport by ambulance (median time 30 vs. 75 min; *p* = 0.040) were associated with shorter OAT (Table 3). Knowledge about the three major stroke criteria together was not associated with OAT (median time 30 vs. 30 min; *p* = 0.460). Correct recognition of one-sided weakness and/or sensory loss was associated with shorter OAT (median time 30 vs. 60 min; *p* = 0.046). Patients who interpreted their situation as a stroke had a shorter median OAT than those without correct interpretation (median time 15 vs. 45 min; *p* = 0.003).

**Table 3:** Associations between socio-demographic, clinical characteristics, elements of help seeking behavior and OAT

Socio-demographic, clinical characteristics		No./No. evaluated (%)	Median OAT (min)	*p* value
Gender	Male	75/142 (52)	30	0.732
	Female	67/142 (47)	30	
Age in years	<50	7/142 (5)	5	0.451
	51–60	22/142 (16)	30	
	61–70	28/142 (20)	43	
	71–80	56/142 (39)	43	
	81–90	23/142 (16)	30	
	>91	6/142 (4)	60	
Stroke in history	Yes	46/142 (32)	60	0.397
	No	96/142 (68)	30	
Living together	Yes	91/138 (66)	30	0.066
	No	47/138 (34)	60	
Diagnosis	Ischemic stroke	126/142 (89)	30	0.475
	Hemorrhage	12/142 (8)	23	
	TIA	4/142 (3)	135	
Localization	Left hemisphere	65/140 (46)	50	0.167
	Right hemisphere	58/140 (42)	30	
	Posterior circulation	17/140 (12)	30	
Aphasia	Yes	24/132 (18)	43	0.948
	No	108/132 (82)	30	
NIHSS	1–4	90/142 (63)	50	0.003*
	5–15	45/142 (32)	30	
	16–20	6/142 (4)	4	
	>20	1/142 (1)	15	
Stroke at daytime	Yes	24/142 (17)	30	0.002*
	No	118/142 (83)	270	
Bystander present	Yes	91/140 (65)	30	0.096
	No	49/140 (35)	60	
Type of referral	Emergency medical number	39/142 (28)	15	0.004*
	General practitioner	83/142 (58)	50	
	Family	12/142 (9)	90	
	Other	4/142 (3)	23	
Mode of transport	Ambulance	103/140 (73)	30	0.044*
	Own transport	33/140 (24)	75	
	Other	4/140 (3)	68	
Elements of help seeking behavior				
Knowledge of 3 stroke criteria^a^ Recognition one-sided weakness and/or sensory loss	Yes	15/140 (11)	30	0.460
	No	125/140 (89)	30	
				0.046*
	Yes	94/139 (68)	30	
	No	45/139 (32)	60	
Recognition face asymmetry	Yes	17/139 (12)	30	0.654
	No	122/139 (88)	33	
Recognition speech disturbance	Yes	51/139 (37)	30	0.276
	No	88/139 (63)	40	
Interpretation stroke situation	Yes	40/142 (28)	15	0.003*
	No	102/142 (72)	45	

Associations calculated with Mann Whitney U test or Kruskal Wallis test were applicable

*OAT* onset-to-alarm time, *NIHHS* National Institute of Health Stroke Scale

*Statistically significant at *p* < 0.05

^a^3 stroke criteria: one-sided weakness and/or sensory loss, face asymmetry and speech disturbance

## Discussion

In our prospective observational study, a shorter OAT after stroke was associated with correct recognition of one-sided weakness and/or sensory loss, correct interpretation of the stroke situation by the patient, stroke characteristics and logistics of stroke care. This is concordant with other studies [9, 16, 17]. In a qualitative study different aspects of interpretation influenced action or no action [9]. One of these aspects was the presence and influence of another person at stroke onset. Our study showed no relation between the presence and influence of another person and OAT. In the study of Faiz et al. stroke severity, transport by ambulance and lower age were significantly associated with earlier admission [17]. We did not find an association between age and OAT. In contrast, another study reported that older patients were more likely to call the emergency medical number [10]. The crucial role of ambulance services has been described [16]. Not only transport by ambulance, but also items as pre-notification to the receiving hospital and telemedicine-based interaction between the hospital and ambulance are suggested for further reduction in treatment time.

Knowledge about stroke symptoms was not associated with shorter OAT. This is in contrast with other studies [3, 18–20]. In a quantitative study of 113 patients awareness of stroke symptoms and signs was associated with earlier hospital arrival [20]. However, awareness was investigated with a structured questionnaire. In our study knowledge was investigated open-ended, for patients could name any symptom they thought about. We showed that recalling stroke symptoms is much easier instead of open-ended questioning. The definition of knowledge about stroke symptoms depends on the research method. In another study with 150 stroke patients, different cognitive aspects were associated with delay [3]. Not only poor knowledge of stroke symptoms, but not realizing the importance of these symptoms resulted in delay. It seems contradictory that knowledge was not associated with OAT in our study, while a correct interpretation of the stroke situation was. However, the interpretation in the setting of an acute stroke situation is different from evaluating knowledge by reproducing single stroke symptoms in the office.

Our findings show that help seeking behavior after stroke is not a fixed process starting with having knowledge about stroke symptoms in general, followed by correct recognition of one’s own stroke symptoms, interpretation as a stroke and finally action. Nearly half of our patients with a stroke undertook no action within the first hour after symptom onset, despite having knowledge or recognizing symptoms. On the other hand, many patients still undertook action without knowledge, incorrect recognition and incorrect interpretation (shown in Fig. 1). A qualitative study proposed a model of help seeking behavior at the time of stroke [9]. Patients seemed to follow fixed steps from knowledge of stroke symptoms towards action in the process of help seeking. Our study does not support this hypothesis. Additional factors, for instance fear, ignorance, ideas about seriousness or access to medical services, may play a role in the decision to seek help [21]. Many studies focused on some isolated factors responsible for patient delay. Demographic factors [22, 23], logistic factors [16, 17] and social or psychological factors [24–27] were studied. All these elements are of influence and may interact with each other, but a complete insight in the process of help seeking behavior has not been found yet. We believe a more complete insight is needed for more successful stroke campaigns. Differentiating the focus and strategies of these campaigns could reach and educate more future stroke patients. Reaching different age groups or people with different education and interest in information should probably be addressed to in different ways. Changing behavior of people with fear for hospitals or denial to be ill are other important elements to be taken into account.

Our study had several limitations. Firstly, our study was performed within a randomized clinical study [13]. The inclusion criteria used in this study led to selection bias. Patients had to be included within 24 h after symptom onset. This will lead to underrepresentation of patients with very long delays in help seeking behavior. Some patients are waiting for days and sometimes even for weeks before consulting a doctor [4, 17]. Secondly, included patients were interviewed after stroke onset, which may have caused a bias towards a better knowledge of stroke symptoms. Recall bias may cause overestimation of stroke symptoms knowledge. Studies have been performed in control populations to assess knowledge of stroke [28, 29]. These studies give insight in knowledge, but no information about the process leading to help seeking in a real stroke situation, in which social and emotional factors may play a decisive role [11, 22, 30, 31]. Patients with aphasia or a language barrier were not included, possibly leading to selection bias. Due to investment of extra time for completing the questionnaire and other logistical factors, not all patients eligible for this sub-study could be interviewed. However, we believe that our results are still generalizable to the overall stroke cohort, because the patients were randomly included. Finally, we investigated help seeking action within the first hour after stroke onset. Over the past 5 years, much effort has been made in shortening the onset to treatment time [16, 32, 33].

## Conclusion

A shorter OAT after stroke is associated with correct recognition of one-sided weakness and/or sensory loss, correct interpretation of the stroke situation by the patient, stroke characteristics and logistics of stroke care, but not by knowledge of stroke symptoms. Our data support the assumption that help seeking behavior after stroke is not a fixed process, starting with having knowledge and followed by correct recognition and interpretation. This process is complex and seems influenced by many factors. Future research must focus on the type of behavior and its motivation when experiencing a stroke. This is crucial to design appropriate stroke campaigns for changing patient behavior after stroke and thereby reducing patient delay optimally.

## Additional files

Additional file 1: Questionnaire. (PDF 71 kb)
Additional file 2: Table S1. Recognition. (PDF 83 kb)
Additional file 3: Figure S1. Face asymmetry and speech disturbance. (PDF 424 kb)

## Abbrevations

IQR: Interquartile range
NIHHS: National Institutes of Health Stroke Scale
OAT: Onset-to-alarm time
PASS: Preventive Antibiotics in Stroke Study
