# Supplementary material for: Help seeking behavior and onset-to-alarm time in patients with acute stroke: sub-study of the preventive antibiotics in stroke study
Source: BMC Neurol. 2016 Nov 25;16:241. doi: 10.1186/s12883-016-0749-2 (PMC5123223; doi:10.1186/s12883-016-0749-2)
Supplement: Additional file 1: — Questionnaire. (PDF 71 kb) [file 12883_2016_749_MOESM1_ESM.pdf]

Appendix. Questionnaire

**General**	
Questions answered by	Patient/ relative/ both
Diagnosis	Ischaemic/ haemorrhagic stroke/ TIA
Right/left-handed	Right/ left
Aphasia present	Yes/ no
Stroke type	Ischaemic/ haemorrhagic stroke
Stroke location	Right/ left hemispheric/ cerebellar
Living alone	Yes/ no
Time until call for help	Minutes/ hours
**Symptoms**	
Time of stroke onset known	Yes/ no/ wake-up
Place at symptom onset	At home/ at work/ other
Way of symptom onset	Acute/ gradual/ unclear
Evolvement before hospital	Increasing/ stable/ decreasing/ fluctuating
**First reaction**	
What was your first reaction	Nothing/ going to bed/ waiting until symptoms disappeared/ contact relatives/ contact alarm number/ contact general practitioner/ other reaction, namely
If not seeking help, what was the reason	Not realizing something was wrong/ thinking symptoms will disappear/ in the past the same symptoms spontaneous disappearing/ unable to call for help/ other reasons, namely
**Bystanders**	
Bystanders present	Yes/ no
Symptoms recognized by bystanders	Yes/ no
Relatives are known with stroke symptoms	Yes/ no/ no idea
**Knowledge about stroke**	
What did you think that happened	Stroke/ no idea/ other, namely
Which symptoms did you have	…
Which stroke symptoms did you know before you had a stroke	No idea/ unilateral facial weakness/ disturbed speech/ unilateral paresis/ unilateral disturbed sensation/ disturbed vision/ decreased consciousness/ headache/ dizziness/ confused/ nausea or vomiting
Which of the next symptoms are stroke symptoms	Weakness or decreased sensation in face, arm or leg/ difficulty with speaking/ visual problems with one or both eyes/ dizziness/ unusual, severe headache
Is treatment of stroke possible	Yes, namely/ no/ no idea
**Transport**	
Referral to hospital by	General Practitioner/ relatives/ self/ went to EMS
Transportation to hospital by	Ambulance/ own transportation/ other
**Time**	
Date and time of onset	dd / mm / yyyy, Hours ; minutes
Date and time calling for help	dd / mm / yyyy, Hours ; minutes
Date and time ambulance arrival	dd / mm / yyyy, Hours ; minutes
Date and time of IVT	dd / mm / yyyy, Hours ; minutes

TIA: Transient Ischemic Attack, EMS: Emergency Medical Services, IVT: Intravenous Thrombolysis
