# Supplementary material for: Help seeking behavior and onset-to-alarm time in patients with acute stroke: sub-study of the preventive antibiotics in stroke study
Source: BMC Neurol. 2016 Nov 25;16:241. doi: 10.1186/s12883-016-0749-2 (PMC5123223; doi:10.1186/s12883-016-0749-2)
Supplement: Additional file 2: Table S1. — Recognition. (PDF 83 kb) [file 12883_2016_749_MOESM2_ESM.pdf]

**Supplement I. Recognition[#] N = 157***	N
**Speech**	
Not being able to speak	13
Difficult, or worsened speaking	24
Strange speaking	2
Blurred speaking	14
Speaking nonsense or inconsistent	2
Trouble with finding right words	3
Babbling	1
Speaking as if drunk	1
Speaking with double tongue	2
Soft speaking	1
Changed speaking	1
Lisping	1
	**65**
**Function**	
Weakness, paralysis, not being able to move, weak or heavy limb (1 sided)	49
Weakness legs, dropping down (2 sided)	2
Not being able to stand	4
Not being able to stand up	5
Not being able to stand on leg, dropping through leg, or knee	5
Not being able to walk	2
Impaired walking	2
Walking as if drunk	1
Strange walking	1
Unstable walking	2
Fuzzy walking	1
Brains uncontrolled	1
	**75**
Dragging foot or leg	5
Foot as if stuck on floor	1
Leg less controlled	1
Unstable leg	1
Strange leg	1
Trembling feet	1
	**10**
Dragging to the left side, hanging over to one side	2
Risk to fall	2
Falling	4
Loosing balance	1
Gliding out of bed	1
	**10**
Impaired function of the hand, not being able to grab something	8
Not being able to use arm	1
Something wrong with the hand	1
Dropping something, dropping something out of the hand	2
Trembling arm	1
Less control over the arm, clumsy hand	2
Impaired fine motorics	1
	**16**
Not being able to open the door with key	1
Impaired typing	1
Not being able to drive the car	1

Not being able to turn around in bed	1
Not being able to hold the rollator	1
	**5**
**Face**	
Face asymmetry	15
Not being able to drink water, fluid running out of mouth	2
Stiff feeling round mouth	2
Hanging lip	1
	**20**
**Vision**	
Diplopia	6
Not being able/ impaired vision	3
Impaired field of vision	3
Not being able to read with one eye	1
Misty vision	3
	**16**
**Sensation**	
No, impaired, or dead feeling	19
Tingling	9
Sleepy arm	1
	**29**
**Dizziness**	16
Dizziness with turning sensation	3
	**19**
**Pain**	
Headache	8
High pressure in head, splashing feeling in head	1
Pain in arms, or legs	2
	**11**
**Malaise**	
Feeling unwell	4
Transpiring	1
Feeling strange in head	2
Feeling strange	1
Feeling as if drunk	2
Feeling snotty	1
Feeling miserable	1
Feeling shaky	1
Nauseous	9
Vomiting	7
	**29**
**Consciousness**	
Non responsive, or loosing consciousness	2
Not reacting	1
Being off-track	2
Impaired notion	1
Impaired orientation	1
Changed behaviour	1
Confused	1
	**9**

**Other**	
Not being able to do a thing	1
Troubled arm	1
Strange movements with arms, or legs	1
Complaints left side of the body	1
Body acting strangely	1
Tinnitus	1
Impaired hearing	1
Problem of the heart	1
Due to physical therapy	1
	**9**
**Nothing**	**1**

[#] More answers per patient were possible
* Missing values: PID 58: neglect? PID 5, 120, 136: no answer given
