# Supplementary figures and images for: Help seeking behavior and onset-to-alarm time in patients with acute stroke: sub-study of the preventive antibiotics in stroke study
Source: BMC Neurol. 2016 Nov 25;16:241. doi: 10.1186/s12883-016-0749-2 (PMC5123223; doi:10.1186/s12883-016-0749-2)

## Supplement II. Process of help seeking behaviour

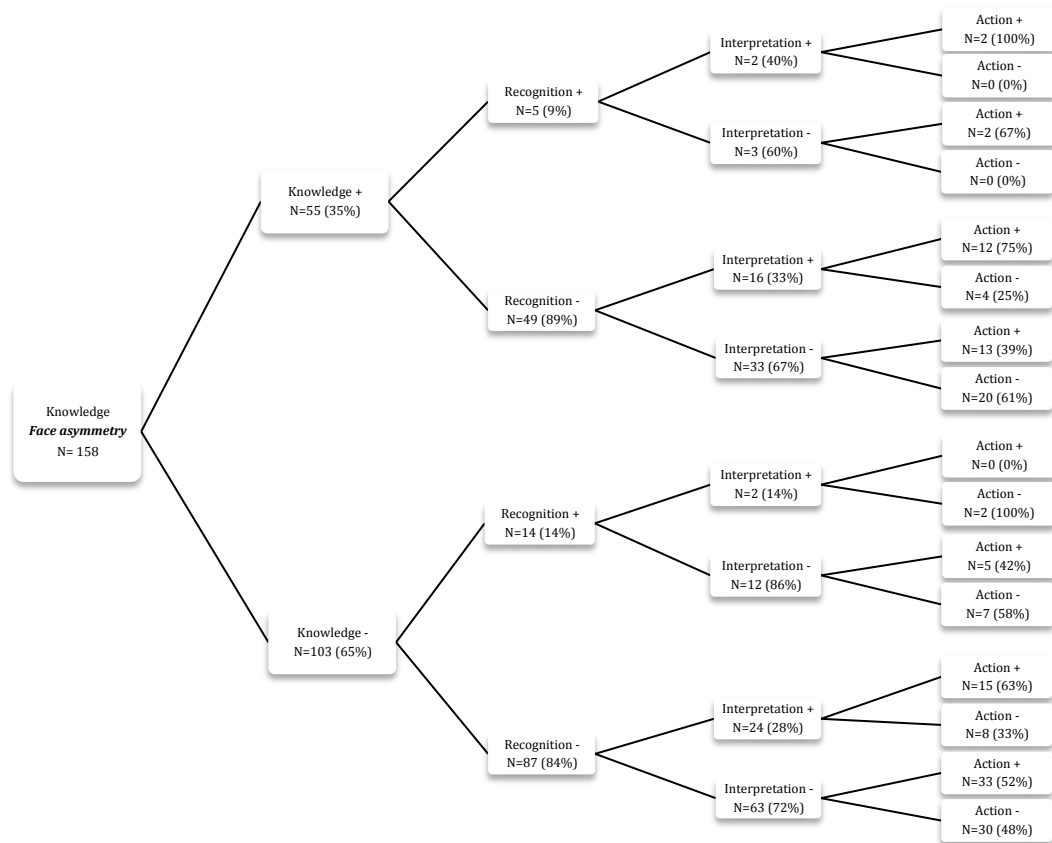

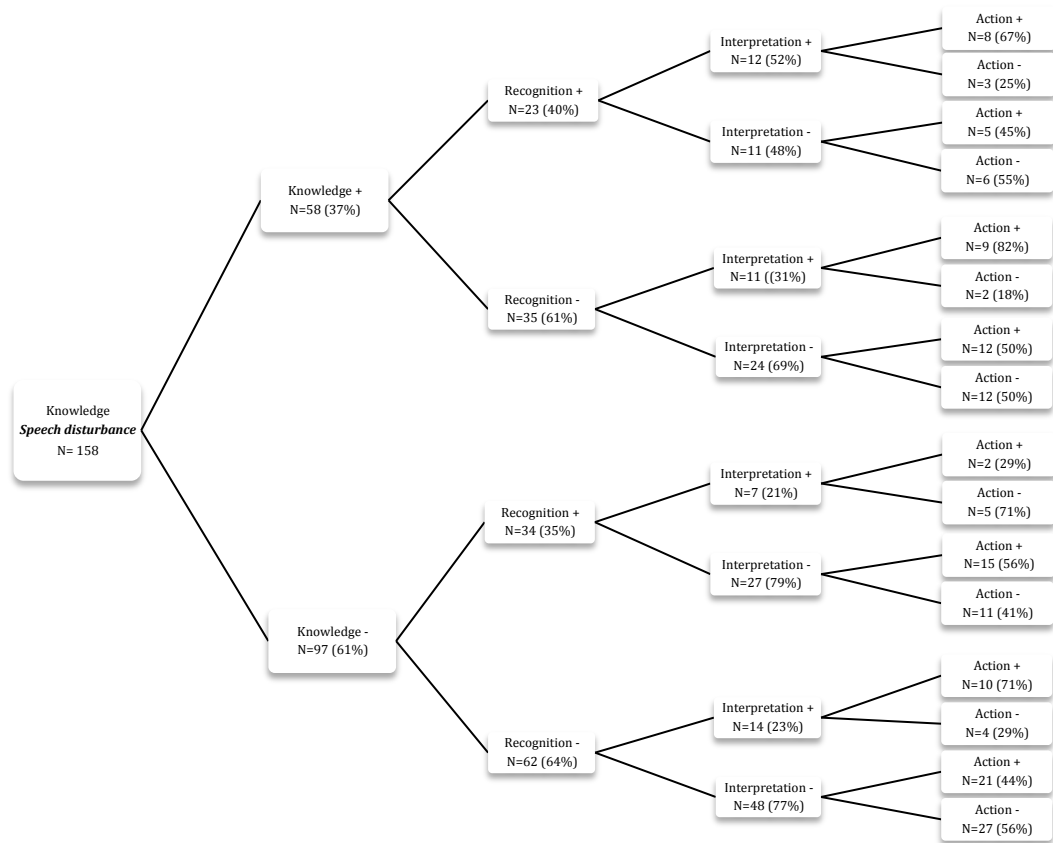

Supplement: Additional file 3: Figure S1. — Face asymmetry and speech disturbance. (PDF 424 kb) [file 12883_2016_749_MOESM3_ESM.pdf]
